# The effect of *Ficus carica* latex on 7, 12-dimethylbenz (*a*) anthracene-induced breast cancer in rats

**Published:** 2018

**Authors:** Fereshte Ghandehari, Mahnoosh Fatemi

**Affiliations:** 1 *Department of Microbiology, Falavarjan Branch, Islamic Azad University, Isfahan, Iran*; 2 *Department of Biology, Falavarjan Branch, Islamic Azad University, Isfahan, Iran*

**Keywords:** Breast cancer, Ficus carica latex, Hematological parameters, Histopathology, Rat

## Abstract

**Objective:**

In traditional medicine,* Ficus carica* (also known as fig) latex is recognized as a remedy with various therapeutic effects. Recently, *in vitro* studies have reported the anticancer effect of this latex on cancer cell lines. This study evaluated the effect of this latex on breast cancer growth, hematological parameters, and histopathology in rats.

**Materials and Methods:**

Twenty-four female rats were randomly divided into 3 groups. In cancerous group, 0.5 ml 7, 12-dimethylbenz (a) anthracene was injected to nipple for breast cancer induction. The control group received sesame oil at the same volume through similar route. In fig latex treated group (Fle), breast cancer was induced and then 0.5 ml of fig latex was intratumorally injected on a daily basis for 4 weeks. Tumor size was measured at the 2^nd^, 4^th ^and 6^th^ weeks of the experiment. Blood samples were used for investigation of the hematological parameters and livers, kidneys and tumor tissues were removed for histopathological analysis.

**Results:**

The tumor size in Fle group was significantly decreased compared to the cancerous group. Haematocrit, hemoglobin, RBC and their indices were significantly decreased, whereas platelet, leukocyte and white blood cell numbers were significantly increased in cancerous group compared to the control group. There were no changes in these parameters in the Fle group compared to the control group. There were severe pathological changes in the livers and kidneys of cancerous group, but not in Fle group.

**Conclusion:**

These results suggest that fig latex could decrease tumor growth without having any adverse effect on hematological and histological factors. However, further investigation is required in this field.

## Introduction

Cancer is one of the main causes of death worldwide. There are various synthetic drugs for cancer treatment; however, many of these agents are usually ineffective and expensive and may cause critical side effects (Zubair et al., 2016 ▶).

Plant products and their derivatives are generally cheaper and safer compared to synthetic drugs and could be used for treatment of different diseases (Grabley and Thiericke, 1999 ▶).Thus, despite wide use of synthetic drugs, herbal medicines are still administered. Many pharmacologists try to produce new drugs from plant products, especially, anticancer drugs (Sa and Das, 2008 ▶).

Plant latex as secondary metabolite is secreted from laticifer cells of about 12000 plant species and contains various amounts of proteinases, alkaloids, tannins, polyphenols, and hydrolytic enzymes, which prevent the growth and hatching of insects’ eggs. Moreover, antibacterial, antiviral, and antifungal potentials of different latex have been verified (Chavan et al., 2015 ▶).

The latex secreted from young leaves of fig (*ficus carica*) has a high medicinal value in Persian traditional medicine as a remedy for many conditions. This latex contains 6-*O*-Oleyl-β-D-glucosyl-β-sitosterol, 6-*O*-linoleyl-β-D-glucosyl-β-sitosterol, caoutchouc, cerin, albumin, resin, 6-*O*-palmitoyl-β-D-glucosyl-β-sitosterol, rennin, sugar, malic acid, and proteolytic enzymes such as, diastase, esterase, lipase, catalase and peroxidase (Rubnov et al., 2001 ▶). It is recommended in the Canon, the book written by Avicenna to consume fig latex in order to treat a wide range of diseases including warts, hypoglycemia, and papillomatosis, and to expel parasitic worms (Patil and Patil, 2011a ▶).

Recently, the anticancer effect of fig latex has been proven against esophageal cancer cell lines (Hashemi and Abediankenari, 2013 ▶), Burkitt B- cell lymphoma (Rubnov et al., 2001 ▶), human glioblastoma, hepatocellular carcinoma (Wang et al., 2008 ▶), gastric cancer cell lines (Hashemi et al., 2011 ▶) and HeLa cell line (Khodarahmia et al., 2011 ▶).

Unfortunately, few *in vivo* studies have investigated the anticancer effect of fig latex. Thus, in the present research, initially breast cancer was induced in the female rats using 12-7- 7, 12-dimethylbenz (*a*) anthracene (DMBA), then, the effect of intratumoral injection of the fig latex on the size of tumor and hematological and histological parameters was assessed. 

## Materials and Methods

**Preparation of fig latex**

*Ficus carica* latex was collected drop by drop after cutting young leaves of fig trees (in Isfahan in July 2015). A voucher specimen was prepared and deposited (No. 019/002/001) in the Department of Biology, Falavarjan Branch, Islamic Azad University, Isfahan, Iran. Fresh latex was diluted with distilled water at a ratio of 1:1 during the treatment stage. 

**Experimental design**

In this study, 24 female Wistar rats (150±20g) were obtained from animal house of the Islamic Azad University, Falavarjan branch and kept under controlled conditions at 22±2°C with 45±5% humidity and 12 hr light/12 hr dark cycle. Rats had free access to distilled water and sterilized food, *ad libitum*. All animal experiments were done according to ethical principles approved by the IASP (Rowan, 1995 ▶). Rats were randomly divided into three groups as follow. Control group: In this group, rats were fed with water and food throughout the experimental period and 0.5 ml of sesame oil was injected in their right nipple at the beginning of the test. Cancerous group: After shaving the chest of rats, 0.5ml of DMBA (5 mg of DMBA dissolved in 0.5 ml of sesame oil) was injected into their right nipple. Fig latex-treated group: The breast cancer was induced in these rats in to the same way as the cancerous group and when tumors reached an average diameter of 10 mm, 0.5 ml of fig latex was intratumorally injected on a daily basis for 4 weeks.

**Measuring volume of cancerous tumors**

Tumor volume was determined by measuring the big and small diameters of all tumors at the 2^nd ^(tumors larger than 10 mm in diameter), 4^th ^and 6^th ^weeks of testing with the aid of a caliper. Volumes of tumors were calculated according to this formula: (tumor volume = 0.5 × (small diameter)^2 ^× large diameter) (Attia and Weiss, 1966 ▶).

**Measurement of hematologic factors**

Rats were anesthetized by intraperitoneal injection of diethyl ether at the end of the experiment. The blood samples were collected directly from the heart. After transferring blood samples to specific tubes, white blood cell (WBC), red blood cell (RBC), hemoglobin (HGB), hematocrit (HCT), mean cell volume (MCV), mean platelet volume (MPV), mean capsular hemoglobin (MCH), red cell distribution width (RDW), platelet (PLT), mean platelet volume (MPV) and platelet distribution width (PDW) were analyzed using an automated hematology analyzer XE-5000 (Sysmex Corporation, Kobe, Japan).

**Preparation of tissue slides for histopathological studies**

After collecting blood samples, the rats were killed by cervical dislocation. Livers, kidneys, and tumors were removed and rapidly fixed in 10% neutral buffered formalin for 48 hr. The tissues were then dehydrated through a 70, 80, 90, 95, and 100% ethanol series. Then, samples were cleared by xylene. Samples were then treated with molten paraffin wax. Paraffin blocks were made of tissues and 5µm slices were prepared by microtome. Paraffin sections were stained with hematoxylin and eosin (H & E) (Abdelhalim and Jarrar, 2012 ▶) viewed under a light microscope. 

**Statistical analysis**

Data were expressed as mean±SD and analyzed by independent-samples *t*-test to determine the size of tumors and One-Way Analysis of Variance (ANOVA) for analysis of other data. The analyses were performed using SPSS 16.0 software. The significance level was set at p≤0.05. 

## Results

**The effect of fig latex on the tumor volume**

There was a significant reduction (p≤0. 001) in the tumor volume in fig latex-treated rats at the 4^th ^and 6^th^ weeks of the experiment compared to the volumes of tumors in rats of cancerous group (Table 1).

**Table 1:** Comparison of the tumor volumes at the 2^nd^, 4^th ^and 6^th^ weeks of the experiment in cancerous and fig latex-treated groups

**Tumor volume (mm** ^3^ **)**	**Cancerous **	**fig latex-treated group **
**2** ^nd ^ **week**	87.79±8.02	97.87±11.30
**4** ^th^ ** week**	267.26±58.70	98.02±4.23***
**6** ^th^ ** week**	482.59±90.58	109.75±10.58***

*** p≤0.001 indicates a statistically significant difference in tumor volume compared to cancerous group. Values are presented as mean±SD.

**Hematological findings**

Hematological parameters in the cancerous group and fig latex-treated group were compared with those of the control group (Table 2). The total WBC count indicated a significant increase in cancerous group compared to the control group. The differential count of WBC showed that the number of lymphocytes significantly increased while the increases in the numbers of basophils, neutrophils, eosinophils and monocytes were not significant. In the cancerous group, platelets counts increased, but mean platelet volume and platelet distribution width were significantly decreased. The total RBC count, the hemoglobin content, hematocrit, mean cell volume, and mean capsular hemoglobin were significantly decreased. However, red cell distribution width indicated a significant increase in cancerous group compared to the control group. No significant difference in hematological factors was observed between rats exposed to fig latex and rats of control group. 

**Histopathological findings**

**Figure 1:**
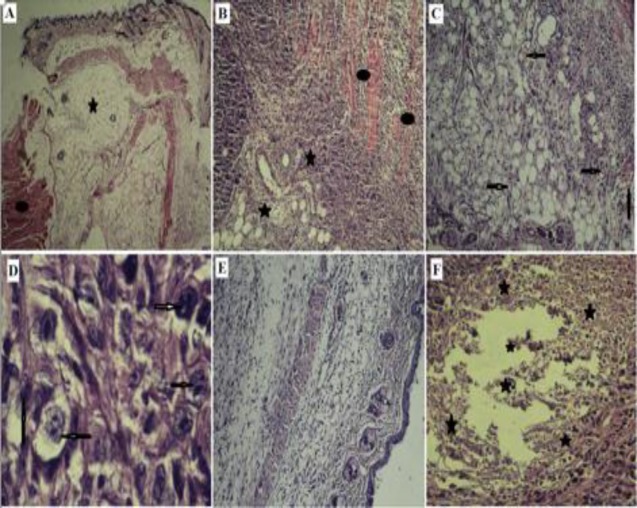
Hematoxylin and eosin (H&E)-stained sections of breast tissue. A: normal fatty (oval) and muscle tissue (star) in control group. B: stromal (oval) and muscular (star) invasion in cancerous group. C and D: Arrows indicate angiogenesis and mitotic features, respectively in cancerous group. E: Breast tissue in the fig latex-treated group. F: Stars indicate necrosis and inflammatory cells in the fig latex-treated group. Magnification: panel A (X40), panels B, C, E, and F (X100) and panel D (X1000).

**Figure 2:**
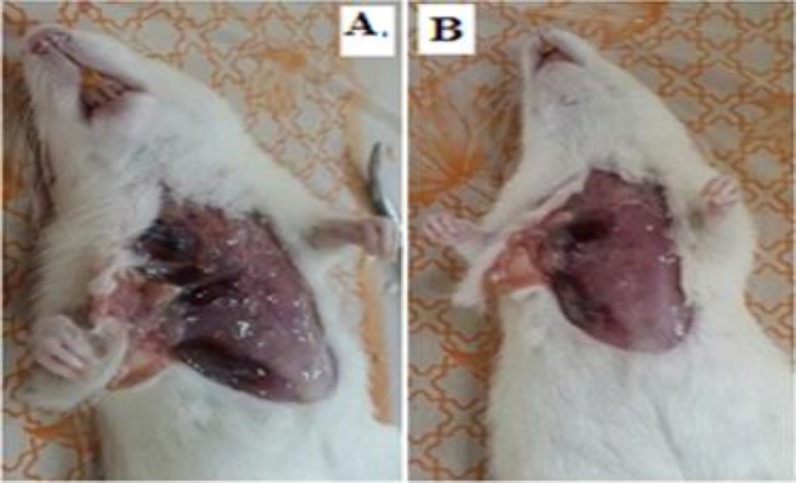
Cancer metastasis in the cancerous group (A). Non metastatic cancer in the fig latex-treated group (B)

The H & E staining indicated breast tissue with normal fatty cells and muscle tissue in the control group rats **(**Figure 1A). Figures 1 B, C & D represent stromal and muscular invasion, angiogenesis and the number of mitotic cells, respectively, in the cancerous group. Figure 1E shows the stromal invasion in fig latex-treated group. Also, large scale necrosis accompanied by infiltration of inflammatory cells was indicated in this group (Figure 1 F). Figure 2A indicates liver metastasis in rats of cancerous group, while tumors in fig latex-treated group were not spread to the livers (Figure. 2B).

**Table 2:** Hematological parameters (mean±SD) of control, cancerous and fig latex-treated groups at the end of the experiment

**Hematological parameters**	**Control group **	**Cancerous group **	**Fig latex exposed group**
**PLT (10** ^3^ ** µl** ^-1^ **)**	727.67±140.34	984.17±188.99*	746.96±96.65
**MPV (fL)**	6.70±0.23	5.82±0.16***	6.40±0.36
**PDW (fL)**	15.00±0.21	14.23±0.56**	14.82±0.28
**RBC (10** ^6^ ** µl** ^-1^ **)**	7.07±0.53	5.62±0.59*	6.22±1.27
**HGB (g dl** ^-1^ **)**	13.18±1.06	11.58±0.83*	12.80±0.87
**HTC (%)**	44.55±4.94	29.40±5.22***	38.64±1.58
**MCV (fL)**	64.38±2.49	47.11±3.98***	61.05±3.38
**MCH (pg)**	19.10±0.58	16.29±1.03***	18.42±0.12
**RDW (%)**	26.22±1.26	41.82±11.16**	32.05±6.09
**WBC (10** ^3^ ** µl** ^-1^ **)**	7.80±1.34	10.68±1.83**	8.46±1.14
**Neu (10** ^3^ ** µl** ^-1^ **)**	0.43±0.18	0.79±0.36	0.43±0.23
**Lym (10** ^3^ ** µl** ^-1^ **)**	1.62±0.71	3.97±1.1***	2.40±0.77
**Mon (10** ^3^ ** µl** ^-1^ **)**	0.99±0.37	1.12±0.38	0.99±0.26
**Bas (10** ^3^ ** µl** ^-1^ **)**	0.003±0.00	0.005±0.002	0.003±0.001
**Eos (10** ^3^ ** µl** ^-1^ **)**	0.003±0.0001	0.008±0.004	0.005±0.002

* p≤0.05,

** p≤0.01, and

*** p≤ 0.001 indicate statistically significant differences compared to control group.

**Figure 3:**
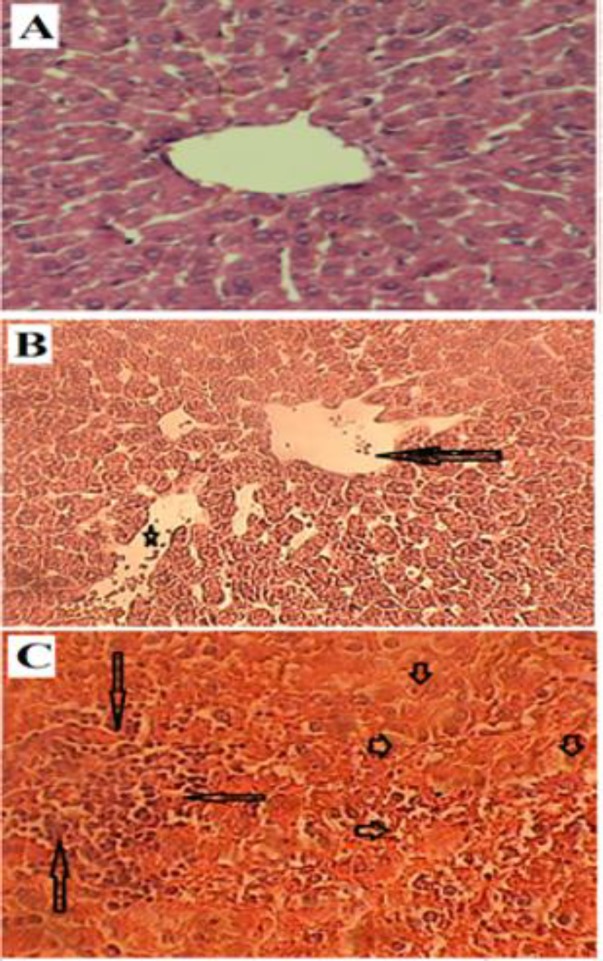
Histological alterations in the livers of rats. (A): control group with normal hepatic lobule and hepatocytes surrounding a central vein. (B): cancerous group with dilated congested liver sinusoids (star) and central vein disarray (arrow). (C): cancerous group with necrotic hepatocytes (short arrows) and inflammatory cells infiltrating into liver lobules (long arrows). (X400)

The histology of the hepatocytes, central veins and sinusoids diameter was normal in livers of control group (Figure. 3A). However, in the livers of the cancerous group, hepatocytes disarray with dilated congested central vein and liver sinusoids (Figure 3B), degeneration of liver hepatocytes and infiltration of inflammatory cells in the hepatic lobules (Figure 3C) were observed.

**Figure 4:**
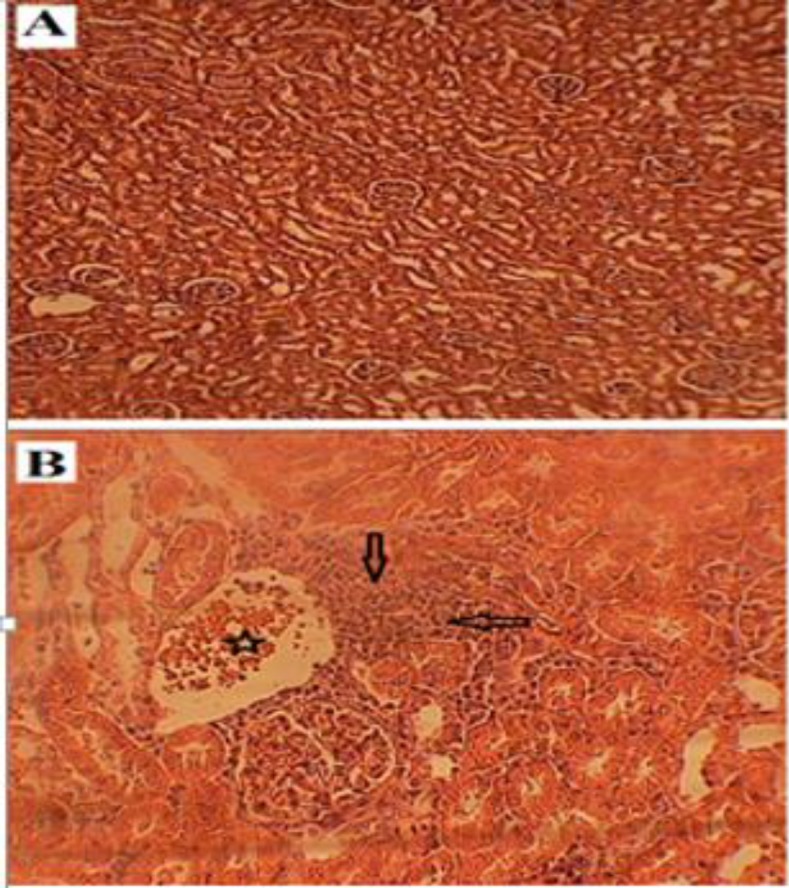
Histological alterations in the kidneys of rats. (A): normal renal tubes and Bowman's capsule in control group (X100). (B): cancerous group with dilated congested of the renal blood vessels (star) and periglomerular and interstitial aggregations of inflammatory cells (arrows). (X400)

Figure 4B shows histopathological changes including periglomerular and interstitial aggregations of inflammatory cells and dilation and congestion of blood vessels in the kidneys of the cancerous group rats. No histological disorder was observed in cortical or central parts of the kidneys of the control group rats (Figure 4A). It was noticeable that after intratumoral injection of the fig latex, histopathological insults were not observed in the livers and kidneys (images not shown) compared with the control (non-fig latex treated) group rats. 

## Discussion

Recently, some *in vitro *studies have shown that fig latex possesses a group of proteases such as ficin, caseinolytic, and gelatinolytic enzymes that induce apoptosis in cancer cells without causing any side effect on normal cells (Rubnov et al., 2001 ▶; Khan et al., 1979 ▶). Therefore, we evaluated the effect of fig latex on cancer cells in an animal model of breast cancer. In the present study, the inhibitory effect of fig latex against the growth of cancerous tumors was observed by comparison of the mean volumes of tumors between the fig latex-treated and cancerous group at the 2^nd^, 4^th ^and 6^th^ weeks of the experiment. Evaluation of tumor tissues also showed extensive necrosis and decreases in the number of mitotic features in fig latex-treated group compared to the cancerous group.

In an old study, Ullman (1952) ▶ confirmed that fig latex inhibits the growth of transplanted and spontaneous tumors. It is well known that escaping from apoptosis and failure in any phase of the cell cycle are characteristics of cancer cells (Sa and Das, 2008 ▶). So, it is likely that induction of apoptosis by fig latex and interaction of this latex with cell cycle have reduced cell division and decreased tumors sizes. 

Wang et al. (2008) ▶ exposed human glioblastoma, hepatocellular carcinoma, and normal liver cell lines to fig latex. They observed reductions of cell proliferation, increases in the number of apoptotic cells, rises of G_0_/G_1_phase cell population, and decreases in S and G_2_*/*M phase cell population.

Khodarahmi et al. (2011) determined the viability of the HeLa cells by reduction of 3-(4, 5-dimethylthiazol- 2-yl)-2, 5-diphenyl tetrazolium bromide (MTT) from formazan following 48-hr incubation with different concentrations of the fig latex and reported that fig latex reduced the HeLa cells survival rate. Hashemi et al. (2011 & 2013) also demonstrated suppressive effect of *F. carica* latex on stomach and esophageal cancer cells line by MTT assay. 

A number of researchers showed that palmitoyle derivative of 6-*O*-Oleyl-β-D-glucosyl-β-sitosterol is the most potent anticancer agent present in *F. carica* latex (Rubnov et al., 2001 ▶). Tezcan et al. (2015) ▶ analyzed the effect of *F. carica* latex on the proliferative and invasion of glioblastoma multiforme cell lines (GBM) using the WST-1 assay and the chick chorioallantoic membrane assay, respectively. They suggested that protocatechuic acid derived from latex might be partially responsible for induction of cell death and inhibition of invasion in this cell line. We believe that phenolic compounds in fig latex are the main inhibitors of tumor growth. In future studies, this possibility will be assessed.

In our investigation, liver metastasis was also observed in rats of cancerous group; while fig latex prevented from secondary breast cancer in other tissues. 

Several researchers reported that the platelets protected cancer cells from the immune system and they exacerbated metastasis by producing fibrin that caused connection of cells to the endothelium of vessels (Mutlu et al., 2012 ▶; Rickles and Edwards, 1983 ▶).

In this research, a significant increase in the number of platelets and a significant decrease in the MPV and PDW were observed in cancerous group compared to control group. MPV and PDW are early indices of platelet activation. Yu et al. (2017) ▶ in a study on 280 patients with thyroid cancer observed that the patients had lower MPV and higher PDW compared to control subjects. 

Since systemic hemostasis and thrombosis activation have been implicated in tumor progression and metastasis (Tanaka et al., 2016 ▶), our findings indicated that cancer inhibited platelet activation. 

In line with our investigation, it has been reported that among patients with lung cancer, the values of coagulation factors such as fibrinogen and PLT were significantly higher in metastatic group compared to non-metastatic group (Liu et al., 2016 ▶).

In our study, no significant difference was observed in the MPV, PDW and PLT in rats exposed to fig latex compared to control group. Platelets play a key role in inflammation and angiogenesis, because these cells are activated by cancer cells and release cytokines, chemokines and angiogenesis mediators that promote both inflammation and angiogenesis (Johnson et al., 2016 ▶).

Therefore, we speculated that fig latex prevented from increases in the number and activity of the platelets due to its anti-angiogenesis and anti-inflammatory effects. The anti-inflammatory activity of fig latex may be associated with its antioxidant properties due to its polyphenols and flavonoids content (Bouyahya et al, 2016 ▶). The anti-angiogenesis and anti-inflammatory effects of *F. carica *demonstrated by a significant decrease in the production of tumor necrosis factor alpha, prostaglandin E2 and vascular endothelial growth factor after administration of *F. carica *leaves extract into the inflamed site of air pouches in a rat air pouch model of inflammation (Eteraf-Oskouei et al., 2015 ▶). Moreover, Mostafaei et al. (2011) ▶ reported that fig latex inhibited the proliferation of endothelial cells of central vein cultured in a three-dimensional medium with collagen matrix and suppressed the production of capillary tubules from these cells without damaging them.

In the present investigation, induction of breast cancer in rats led to significant decreases in RBC, HGB, HCT, MCV, and MCH but caused a significant increase in RDW that is a sign of anemia. 

Ay et al. (2015) ▶ measured RDW and MCV in 115 patients with colon polyps and 30 with colon cancer. They observed that RDW of patients with colon cancer was significantly higher than that of the patients with colon polyps. No significant differences were detected in MCV between two groups. In a similar study, a significant increase in RDW and a significant decrease in MCV were observed in patients with colon cancer (Wspell et al. 2004 ▶).

Anemia is one of the most common side effects caused by inflammation, infection and neoplastic diseases which are called anemia of chronic diseases (Dolan et al., 2010 ▶). It was shown that cancer, via inhibition of erythropoietin secretion causes reduced survival and proliferation of erythroid progenitor cells in special areas of the liver and kidney that decreased erythrocytes lifetime and induced anemia (Duncan et al., 1994 ▶).

There were no statistically significant differences in the total RBC count and the related factors between the fig latex-treated group and the control group. It may be due to anti-inflammatory effect of the fig latex (Lansky et al., 2008 ▶), that has prevented the destruction of erythropoietin-secreting tissues and accordingly, prevented a decrease in RBC and blood cell indices.

The possibility of the induction of inflammation was assessed by comparing the number of immune cells between the cancerous group and the control group.

WBC, lymphocytes, monocytes, basophils and eosinophils counts were higher in cancerous group compared to control group; this increase was significant only in the numbers of lymphocytes and WBC that can be due to the induction of inflammation in this group. Epidemiological studies have shown that in some cancer types, any oncogenic change induces an inflammatory microenvironment that stimulates development of tumors (Mantovani et al. 2008 ▶)

Granger and Kontoyiannis (2009) ▶ observed leukocytosis in 758 patients with non-hematologic cancer and observed the occurrence of secondary infections in their bodies following cancer. Moreover, several studies have shown a positive relationship between white blood cell count and risk of cancer mortality (Shankar et al. 2006 ▶; Erlinger et al. 2004 ▶; Grimm et al. 1985 ▶).

After intratumoral injection of the fig latex, no significant changes were seen in the total WBC count in the fig latex-treated group compared to the control group.

While fighting against infections, immune system cells generate free radicals which may eventually induce oxidative stress in the body. Administration of antioxidants in many chronic diseases, including cancer might fight against side effects of oxidative stress by improving antioxidant defense system (Knight, 2000 ▶). Thus, it seems that antioxidant compounds in fig latex including flavonoids and polyphenolic compounds (Mahmoudi et al., 2016 ▶) inhibited induction of the oxidative stress and inflammation and subsequently prevented an increase in WBC. 

In the current investigation, histopathological examination revealed that cancer led to destruction of hepatocytes, dilation and polycythemia at central veins and sinusoids as well as focal accumulation of mononuclear cells among hepatic lobules in the liver and a disorder in renal tubular, dilation and congestion of blood vessels as well as accumulation of inflammatory cells in the kidney. These changes obviously indicated tissue inflammation. 

In line with our investigation, Jonkers and Derksen (2007) ▶ observed renal deficiency, acute inflammation and necrosis of renal tissue after induction of breast cancer metastasis in mice. Structural changes in hepatic lobules and aggregations of inflammatory cells in central vein were also observed in the study of Russo and Russo (1996) ▶ following induction of cancer.

No abnormal tissue change was observed in the livers and kidneys of the fig latex-treated rats compared to control group that can be attributed to strong antioxidant and anti-inflammatory effects of fig latex (Lansky et al., 2008 ▶; Patil and Patil, 2011b ▶; Jasmine et al., 2015 ▶) and its inhibitory effect on tumor growth (Ullman,1952 ▶).

In the present research, administration of fig latex prevented the oversized growth of cancerous tumors without causing side effects on hematologic parameters and induction of the inflammation in tissues that can be attributed to antioxidant, anti-inflammatory and pro-apoptotic effects of this natural product.
